# Emergence and epidemiology of dominant variants of human metapneumovirus in the United States between 2016 and 2021

**DOI:** 10.1128/mbio.02619-25

**Published:** 2026-01-12

**Authors:** Lora Lee Pless, Lambodar Damodaran, Ray Pomponio, Rose Patrick, Marissa Pacey Griffith, Sara Walters, Kady D. Waggle, Atalia Pleskovitch, Vatsala Rangachar Srinivasa, Cole A. Varela, Lee H. Harrison, John P. Barton, Louise H. Moncla, Marian G. Michaels, John V. Williams, Anna F. Wang-Erickson

**Affiliations:** 1 Microbial Genomics Epidemiology Laboratory, Center for Genomic Epidemiology, University of Pittsburgh 6614, Pittsburgh, Pennsylvania, USA; 2 Division of Infectious Diseases, University of Pittsburgh School of Medicine 271847, Pittsburgh, Pennsylvania, USA; 3 Department of Pathobiology, School of Veterinary Medicine, University of Pennsylvania 634332, Philadelphia, Pennsylvania, USA; 4 Division of Infectious Diseases, Department of Pediatrics, University of Pittsburgh School of Medicine 271847, Pittsburgh, Pennsylvania, USA; 5 Department of Epidemiology, School of Public Health, University of Pittsburgh 171669, Pittsburgh, Pennsylvania, USA; 6 Department of Computational and Systems Biology, University of Pittsburgh School of Medicine 196206, Pittsburgh, Pennsylvania, USA; 7 Department of Pediatrics, University of Wisconsin-Madison School of Medicine and Public Health 200763, Madison, Wisconsin, USA; Rutgers-Robert Wood Johnson Medical School, Piscataway, New Jersey, USA

**Keywords:** human metapneumovirus, whole genome sequencing, epidemiology, phylogenetic analysis, pediatric infectious disease

## Abstract

Human metapneumovirus (HMPV) causes acute respiratory disease worldwide and is the second leading cause of lower respiratory infection and hospitalization in young children in the USA. There is no licensed vaccine or therapeutic. HMPV mutates rapidly; however, the specific genomic features that explain strain dominance remain undefined because there is limited routine genomic surveillance of HMPV. We analyzed prospectively collected nasal specimens and medical data from 8,000 pediatric acute respiratory infection cases and sequenced 219 HMPV whole genomes from Pittsburgh, PA, between 2016 and 2021. Only A2, B1, and B2 subgroups were detected. The dominant subgroup varied between seasons. Variants with an in-frame 111- or 180-nucleotide (nt) insertion that nearly duplicates the preceding flanking region in the 660-nt G gene (encodes the attachment protein) were the predominant A2 viruses detected by 2016–2017. Among B2 viruses, variants with smaller in-frame insertions in the same location of the G gene became dominant by 2017–2018. Each insertion length formed a distinct phylogenetic clade. The insertions are in the ectodomain and contain positively charged residues or predicted O-glycosylation sites. Epidemiological analysis revealed that HMPV infection was independently associated with age, insurance type, and comorbidities. Elevated disease severity was independently associated with age and comorbidities, although not with HMPV subgroup. To our knowledge, in the USA, this is the earliest detection of the A2 insertion variants and the first report of the B2 insertion variants. It is the largest population-based genomic HMPV study that provides a detailed phylodynamics and epidemiological analysis of prospectively collected clinical specimens.

**IMPORTANCE:**

Human metapneumovirus (HMPV) is a leading cause of lung infection and pediatric hospitalizations worldwide for which there is no licensed vaccine or therapeutic. Because HMPV mutates rapidly, understanding which mutations enhance its ability to multiply and spread is important for the development of interventions and treatments. We prospectively collected patient data and nasal specimens from children with symptoms of acute respiratory illness. The predominant A2 and B2 HMPV variants circulating in the population contained insertions in the attachment protein, which suggests that these insertions may be advantageous to the virus. Furthermore, our analysis suggests that age, insurance type, and underlying health conditions were associated with HMPV infection. Age and underlying health conditions were associated with elevated HMPV disease severity, whereas HMPV subgroup was not. This large HMPV genomic epidemiological study provides insight into patient factors associated with disease and the emergence of the dominant variants in the USA.

## INTRODUCTION

Human metapneumovirus (HMPV) is a significant global cause of acute respiratory disease in children and adults. Discovered in 2001, HMPV is the second leading cause of pediatric lower respiratory infection and hospitalization, costing $277M per year in the USA alone (1–3). Globally, HMPV was estimated to be associated with 14.2 million acute lower respiratory cases and 643,000 hospitalizations in children under 5 years in 2018 (4). As there is no licensed vaccine or antiviral, most individuals are infected by age 5 years (5, 6). Moreover, reinfections often cause disease in older children and adults that can lead to hospitalization, especially in older adults and those with underlying comorbidities (7, 8). Because asymptomatic HMPV infection is rare, infection almost always causes distressing symptoms, including fever, rhinorrhea, cough, wheeze, otitis media, and pneumonia (2, 8, 9).

HMPV is a single-stranded negative-sense RNA virus that has a 13.3 kb genome with eight genes encoding nine proteins (10). HMPV lineages are organized into four genotypic subgroups: A1, A2, B1, and B2 (11, 12). However, the A1 subgroup has not been detected since 2006 and may be extinct (13, 14). The virus continues to evolve, as evidenced by the emergence of two A2 variants, initially detected in Japan and Spain, that harbor insertions that are near-duplications of 111 and 180 nucleotides (nt) in the G gene, which encodes the surface protein that attaches to host cells during infection (15–17). These variants have since been reported in other countries (14, 18, 19). The 111-nt variant became the dominant A2 strain circulating in Yokohama, Japan, by 2018 and in Barcelona, Spain, by 2021 (20, 21).

Understanding how HMPV is evolving and the predominant variants circulating in the population is critical to the development and assessment of effective future vaccines and therapeutics. However, HMPV is not included on most point-of-care rapid tests, such as those available for other respiratory viruses like influenza or SARS-CoV-2 (22). Clinical testing for HMPV in hospitals is not routinely ordered for all patients presenting with acute respiratory illness (ARI), since HMPV identification would not change the treatment plan, as there is no targeted antiviral for HMPV (22). This limits the availability of systematically collected clinical specimens and the ability to do routine genomic surveillance to monitor how HMPV is evolving. Furthermore, sequencing the full-length HMPV genome has been historically difficult due to regions that are prone to drop out, although recent advancements in sequencing methods optimized for HMPV have greatly improved whole-genome sequencing efficiency and quality (23, 24). Consequently, studies tend to use convenience residual patient specimens, and there are few genomic epidemiological studies of HMPV (14, 19, 21, 25).

We therefore conducted the largest prospective population-based genomic epidemiological study to sequence and analyze 219 full-length HMPV genomes from pediatric specimens, using data from nearly 8,000 ARI patient medical records between 2016 and 2021 in Pittsburgh, Pennsylvania. Because little is known about the emergence of G insertion variants in the USA, we identified mutations in the G gene, assessed frequency changes over time, and described the evolutionary relationships of these variants in both local and global geographic contexts using publicly available whole-genome sequences. We also identified patient factors associated with HMPV infection and disease severity.

## MATERIALS AND METHODS

### Study participants and data collection

Nasal swab specimens were collected from pediatric patients <18 years with ARI symptoms enrolled in inpatient, emergency department, and outpatient settings during December 1, 2016–August 31, 2021 in Pittsburgh, PA, as previously described (26). Briefly, eligibility criteria included having at least one qualifying ARI sign or symptom and illness duration of <14 days. Inpatients were eligible if enrolled within 48 h of admission. Children were excluded if they were previously enrolled in this study <14 days before the current visit or hospitalization, hospitalized <5 days after a previous hospitalization, hospitalized for a known non-respiratory cause, or had fever and neutropenia from chemotherapy. Parents or legal guardians of eligible children gave informed consent before data collection via a standardized parent/guardian interview, medical chart review, and collection of nasal specimens.

### Specimen collection and viral detection

As previously described, nasal specimens were collected using flocked swabs, which were placed in universal transport medium and stored at 4°C for up to 72 h until media was aliquoted and stored at −80°C. Reverse transcriptase quantitative PCR (RT-qPCR) was used to detect HMPV, respiratory syncytial virus (RSV), influenza, human parainfluenza 1-3 (PIV), rhinovirus/enterovirus, adenovirus, and SARS-CoV-2 (starting in 2020) (26). The study was approved by the University of Pittsburgh Institutional Review Board (STUDY19070206). This study followed the Strengthening the Reporting of Observational Studies in Epidemiology (STROBE) reporting guideline.

### Statistical analyses

Descriptive statistics were used to summarize the demographic and clinical characteristics of ARI cases over the entire cohort with known HMPV status, as well as a sub-cohort with subtyped HMPV samples. An infection was defined as a positive HMPV test result. Prevalence was defined as the number of HMPV infections during the study period per total population of unique patients, and 95% confidence intervals (CIs) were estimated by bootstrap percentiles based on 1,000 samples of the patient cohort. Demographic characteristics included age, sex, race/ethnicity, and insurance type. Clinical characteristics included the presence of pre-existing conditions and viral pathogens detected. In addition to HMPV, detectable viral pathogens included influenza, para-influenza, RSV, rhinovirus, and adenovirus.

Using all ARI cases, we assessed the effect of various factors on HMPV infection status, treated as a dichotomous outcome. We reported unadjusted and adjusted odds ratios (ORs) for each factor from generalized estimating equations (GEEs), treating repeated enrollments for the same child as clustered observations and specifying an exchangeable correlation structure. Focusing on HMPV cases only with available subgroup information, we assessed the effects of both subgroup and A2-insertion size on illness severity while adjusting for selected demographic and clinical factors. Illness severity was treated as a five-level ordinal outcome and modeled using proportional odds regression. Outcome levels were as follows, in increasing order of severity: routine ED/clinic discharge; admission to hospital without oxygen support; admission to hospital with standard supplemental oxygen; admission to the ICU without invasive mechanical ventilation, extracorporeal membrane oxygenation (ECMO), or death; and admission to the ICU with invasive mechanical ventilation, ECMO, or death. Proportional odds regression has been shown to improve statistical power beyond simple logistic regression in observational studies with discrete clinical endpoints, such as hospital outcomes (27). Data analyses and statistical modeling were performed using R version 4.4.1 (28).

### HMPV whole-genome sequencing and assembly

#### RNA extraction and RT-qPCR

Nucleic acid was extracted from 200 µL of transport media and eluted in 90 µL of elution solution using the 5× MagMAX-96 Viral RNA or Viral/Pathogen Nucleic Acid Kit (Applied Biosystems) and MagMAX Express 96 (Applied Biosystems) or King Fisher Apex (Thermo Fisher Scientific) liquid handlers. HMPV detection was determined by RT-qPCR with the AgPath-ID One-Step RT-PCR kit (Applied Biosystems) using the following thermocycling conditions: reverse transcription at 50°C for 30 min, reverse transcriptase inactivation/initial denaturation at 95°C for 10 min, followed by 45 cycles of 95°C for 15 min and 60°C for 30 s.

#### cDNA synthesis and genome amplification

HMPV genomes were amplified using a tiled, overlapping amplicon-based method optimized for whole-genome sequencing of HMPV (24). SuperScript IV VILO Master Mix (Thermo Fisher Scientific) was used to generate cDNA from 4 µL extracted RNA per 20 µL reaction. Platinum SuperFi PCR Master Mix (Thermo Fisher Scientific) was used to amplify each of the four amplicons that spanned the entire genome in separate 25 µL reactions and 5 µL cDNA, using the primers listed in Table S1. The thermocycling conditions were as follows: initial denaturation at 98°C for 30 s; followed by 44 cycles of denaturation at 98°C for 10 s, annealing at 60°C for 20 s, and extension at 72°C for 2 min 10 s; and a final extension at 72°C for 5 min. PCR products were assessed using the TapeStation 4200 and D5000 ScreenTape and reagents (Agilent).

#### Sample pooling and library preparation

The four amplicons per sample were pooled together into a single tube at approximate equimolar concentration and purified using standard Ampure XP beads (Beckman Coulter). Sequencing libraries were prepared using the Illumina DNA Prep kit and unique 10 bp dual indices, with a target insert size of 280 bp. Samples were sequenced on the Illumina NovaSeq X Plus, producing 2 × 151 bp paired-end reads.

#### Bioinformatic quality control analyses

Raw reads were demultiplexed using bcl2fastq (Illumina), adapter sequences were trimmed, and data quality control was assessed using fastp (29). Reads were classified by species using our customized reference database containing publicly available viral genomes from NCBI Virus (date accessed: March 2023) with Kraken 2 (30), followed by removal of reads mapping to the human genome (GRCh38/hg38). Next, *de novo* genome assembly was performed using rnaviralSPAdes (31) per sample, incorporating BLAST to identify the best matching viral genome from our customized viral genome database. Contigs were aligned to the best matching viral genome, and scaffolds were assembled into the genomes using nucmer (32). Nucleotide depth was computed using bowtie2 and bedtools (33, 34). Samples passed our quality control criteria if ≥95% of the genome was covered with at least 10× depth; the median coverage depth for all genomes was 30,433× (range, 6,662–108,777), as assessed using bedtools (34) and CheckV (35). Protein annotations were determined using Prokka (36) and visual inspection.

### Sequence analyses

The HMPV subgroup (A1, A2, B1, or B2) was determined by comparing each sample to reference viral genome sequences using BLAST (A1 subgroup: 00-1 [NC_039199]; A2 subgroup: NL/00/17 [FJ168779]; B1 subgroup: NL/1/99 [AY525843]; and B2 subgroup: NL/94/01[FJ168778]). MAFFT v7.49 (37) was used to make multiple sequence alignments, which were subsequently inspected using Geneious Prime 2025.0.3 (https://www.geneious.com/). A few suspected sequencing errors or assembly anomalies were removed from the final genome sequence as noted in Table S2. G gene insertions were detected by sequence alignment comparison against the appropriate subgroup reference sequence lacking insertions, as listed above. Sanger sequencing was performed as previously described (16) to confirm that our *de novo* genome assembly methods could accurately detect the presence or absence of G gene insertions using a representative sample of 50–100% of each insertion size.

### Phylodynamic analysis

A Bayesian phylogenetic modeling approach was used to characterize the transmission of HMPV in Pittsburgh locally and within the global context. Whole-genome sequences for viruses collected in Pittsburgh from February 2, 2017, to April 20, 2020, were organized into subgroups A2, B1, or B2. Global contextual sequence data and associated metadata, including location and date of isolation, were collected for all available HMPV whole-genome sequences from humans in the NCBI Virus database (date accessed: August 13, 2024) (38). Data were filtered to include only complete genome sequences with associated collection year and geographic location at the country level, available after January 1, 2010 (Fig. S1). Multiple sequence alignment was performed for all collected sequence data separately for each subgroup using MAFFT v7.2, and alignments were visually inspected using AliView v1.28 (37, 39). Because some global sequences did not have metadata for the subgroup of the sample, we performed a maximum likelihood phylogenetic reconstruction using FastTree v2.1.11 and classified sequences into global subgroup data sets based on proximity to sequences with a subgroup label within the same clade (40). G gene insertions in global sequences were identified as described above. A few global sequences that had insertion sizes resulting in frameshifts were annotated on the phylogeny as indicated in Table S3. We then created data sets to perform a local (Pittsburgh) and global analysis. For each local and global analysis, we generated one dataset for each subgroup as well as for all subgroups combined, resulting in eight total data sets (Pittsburgh only—A2, B1, B2, and all subgroups combined; Pittsburgh + global—A2, B1, B2, and all subgroups combined). The resulting data sets had the following numbers of sequences per data set: Pittsburgh only: A2 = 91, B1 = 53, B2 = 75, total = 219; Pittsburgh + global: A2 = 319, B1 = 114, B2 = 157, all = 590. Maximum likelihood phylogenetic reconstruction was performed for each data set using FastTree v.2.1.11, and temporal signal was assessed using TempEst v.1.5.3 (Fig. S2 and S3) (41).

We performed phylogenetic reconstruction using BEAST v1.10.4 for all data sets with the following parameters and priors (42). For all global samples where only the year of collection was provided, we set the date to the middle of the year (i.e., 2016-06-01) and set an uncertainty of half a year to cover the entire year of collection. We chose an HKY nucleotide substitution model with gamma-distributed rate variation among sites and a constant-size coalescent (43, 44). Based on root-to-tip analysis, we chose an uncorrelated lognormal relaxed clock with a uniform ucld.mean prior ranging from 0 to 1 and a mean of 0.005 (45). We ran three independent MCMC chains with a chain length of 10 million states, sampling every 1,000 states, for each data set. Runs were diagnosed using Tracer v1.7.2 to assess adequate effective sampling size across parameters, defined as an effective sample score of at least 200. Tree files were combined and resampled using LogCombiner v1.10.4, discarding burn-in between 10-20%, to create a posterior sample of 10,000 trees. Maximum clade credibility (MCC) trees for each data set were created using TreeAnnotator v1.10.4 with a posterior limit of 0.9 to annotate internal nodes. Phylogenies were visualized using ggtree v3.10.1 (46). All XMLs for BEAST analyses and scripts used for visualization of results can be found in the following GitHub repository: https://github.com/moncla-lab/HMPV-Pittsburgh.

## RESULTS

### Descriptive characteristics of study population

A total of 7,982 ARI cases in 6,945 patients enrolled from December 1, 2016, to August 31, 2021, were tested for HMPV and analyzed in this study (Table 1). The overall prevalence of HMPV infection was 5.5% (379 of 6,945; 95% CI, 5.1–5.9%). The median (IQR) age of ARI cases was 1.75 (0.75, 4.0) years, and 58.2% were male. The majority of ARI cases were under 5 years old (80.3%). Of the 379 HMPV cases, 112 (29.6%) were classified as co-infections, in which HMPV and at least one other respiratory virus were detected. Among co-infected HMPV cases, rhinovirus/enterovirus was the most detected other virus (18.2%), followed by adenovirus (7.8%). HMPV subgroup was determined for 249 specimens, and full-length HMPV genome sequences were obtained for 219 specimens. The characteristics of the HMPV subtyped cases were similar to those of the overall HMPV-positive (HMPV+) cases (Table 1).

**TABLE 1:** Demographic and clinical characteristics by HMPV infection status^*a*^

	Subtyped HMPV cases, *N* = 249^*b*^	Overall, *N* = 7,982^*c*^	HMPV infection status		Unadjusted OR (95% CI)	Adjusted OR (95% CI)
			Negative, *N* = 7,603	Positive, *N* = 379^*d*^		
Age in years, median (IQR)	1.33 (0.58, 3.00)	1.75 (0.75, 4.00)	1.83 (0.75, 4.08)	1.42 (0.58, 3.00)	0.92 (0.89, 0.95)	NI
Age in years (min, max)	[0, 14.8]	[0.0, 17.9]	[0.0, 17.9]	[0.0, 17.8]		
Age group, *n* (%)						
Less than 1 year	91 (36.5)	2,499 (31.3)	2,363 (31.1)	136 (35.9)	2.35 (1.61, 3.43)	2.86 (1.89, 4.35)
1 to <2 years	54 (21.7)	1,685 (21.1)	1,604 (21.1)	81 (21.4)	2.08 (1.39, 3.11)	2.42 (1.58, 3.70)
2 to <3 years	38 (15.3)	987 (12.4)	936 (12.3)	51 (13.5)	2.36 (1.52, 3.67)	2.63 (1.68, 4.13)
3 to <5 years	50 (20.1)	1,241 (15.5)	1,165 (15.3)	76 (20.1)	2.80 (1.86, 4.21)	3.01 (1.98, 4.57)
5 years or older	16 (6.4)	1,570 (19.7)	1,535 (20.2)	35 (9.2)	REF	REF
Sex, *n* (%)						
Female	108 (43.4)	3,333 (41.8)	3,175 (41.8)	158 (41.7)	0.99 (0.80, 1.23)	1.00 (0.80, 1.24)
Male	141 (56.6)	4,649 (58.2)	4,428 (58.2)	221 (58.3)	REF	REF
Race and ethnicity, *n* (%)						
Black non-Hispanic	78 (31.7)	2,513 (31.9)	2,388 (31.8)	125 (33.3)	1.08 (0.86, 1.37)	1.08 (0.84, 1.39)
White non-Hispanic	130 (52.8)	4,262 (54.1)	4,063 (54.1)	199 (53.1)	REF	REF
Other non-Hispanic^*e*^	27 (11.0)	777 (9.9)	740 (9.9)	37 (9.9)	1.06 (0.73, 1.52)	1.03 (0.71, 1.49)
Hispanic	11 (4.5)	329 (4.2)	315 (4.2)	14 (3.7)	0.96 (0.55, 1.67)	0.97 (0.56, 1.70)
Insurance type, *n* (%)						
Private	79 (33.1)	2,815 (36.4)	2,695 (36.6)	120 (33.0)	REF	REF
Public	149 (62.3)	4,770 (61.7)	4,540 (61.6)	230 (63.2)	1.11 (0.88, 1.40)	1.05 (0.82, 1.34)
Both	5 (2.1)	47 (0.6)	41 (0.6)	6 (1.6)	3.26 (1.35, 7.83)	3.07 (1.27, 7.43)
None	6 (2.5)	98 (1.3)	90 (1.2)	8 (2.2)	2.16 (1.02, 4.58)	2.22 (1.05, 4.70)
Any pre-existing condition, *n* (%)^*f*^	100 (40.3)	2,802 (35.1)	2,653 (34.9)	149 (39.4)	1.25 (1.01, 1.55)	NI
Pre-existing condition, *n* (%)						
Chronic lung disease^*g*^	53 (21.4)	1,770 (22.2)	1,687 (22.2)	83 (22.0)	1.01 (0.79, 1.30)	1.25 (0.94, 1.65)
Neurologic/neuromuscular disease^*h*^	24 (9.7)	535 (6.7)	500 (6.6)	35 (9.3)	1.45 (1.01, 2.09)	1.75 (1.19, 2.56)
Immunocompromised condition	3 (1.2)	127 (1.6)	123 (1.6)	4 (1.1)	0.69 (0.26, 1.86)	0.84 (0.31, 2.27)
HMPV cases with multiple viral detections, *n* (%)	59 (23.7)	NA	NA	112 (29.6)	NI	NI
Other viruses detected, *n* (%)						
Adenovirus	16 (6.5)	628 (8.1)	599 (8.1)	29 (7.8)	NI	NI
Influenza	3 (1.2)	655 (8.4)	644 (8.7)	11 (2.9)	NI	NI
Parainfluenza	7 (2.8)	705 (8.8)	695 (9.1)	10 (2.6)	NI	NI
Respiratory syncytial virus	2 (0.8)	1,834 (23.0)	1,824 (24.0)	10 (2.6)	NI	NI
Rhinovirus/enterovirus	36 (14.5)	3,144 (39.4)	3,075 (40.5)	69 (18.2)	NI	NI

^*a*^ OR: (adjusted) odds ratio from logistic regression; CI: confidence interval; IQR: inter-quartile range, 25th to 75th percentiles; NI: not included due to pre-specification and/or redundant variable(s) already in model; REF: reference group, no odds ratio reported; NA: not applicable.

^*b*^ Of 249 cases with known HMPV subgroup, 246 were unique patients (three cases were repeated infections/enrollments). Repeated measures were accounted for in ORs and CIs using generalized estimating equations (GEEs).

^*c*^ Of 7,982 ARI cases analyzed, 6,945 were unique patients (1,037 ARI cases were from patients enrolled more than once during the study period). Repeated measures were accounted for in ORs and CIs using generalized estimating equations (GEEs).

^*d*^ 374 HMPV cases were from unique patients. Five patients had two HMPV detections each during the study period, which likely represented independent infection events. The two HMPV detections per patient were either separated by at least 10 months or belonged to different HMPV subgroups. None of the five patients were immunocompromised.

^*e*^ Patients who indicated Asian, Asian American, American Indian, Alaska Native, Native Hawaiian, Pacific Islander, or more than one category were classified as other race due to small sample sizes.

^*f*^ Includes cardiovascular disease, chronic kidney disease, Down syndrome, genetic/metabolic disorders, blood disorders, chronic liver disease, diabetes mellitus, chronic endocrine conditions, chronic lung disease, congenital heart defects, neurologic/neuromuscular disease, and immunocompromised status.

^*g*^ Includes reactive airway disease, chronic lung disease of prematurity, cystic fibrosis, bronchopulmonary dysplasia, and unspecified other lung diseases.

^*h*^ Includes cerebral palsy, seizure disorders, and unspecified other neurologic or neuromuscular disorders.

### Factors associated with HMPV infection and elevated disease severity

Age, insurance type, and neurologic/neuromuscular disease were each associated with HMPV infection after adjusting for other characteristics (Table 1). Compared to school-aged children ≥5 years, all younger age groups had at least twice increased odds of HMPV infection (e.g., infants [<1 year] AOR, 2.86; 95% CI, 1.89–4.35). Compared to children with private insurance, those with no insurance had twice higher odds of HMPV infection (AOR, 2.22; 95% CI, 1.05–4.70), and those with both public and private insurance had three times higher odds of infection (AOR, 3.07; 95% CI, 1.27–7.43). Children with neurologic/neuromuscular disease had 75% increased odds of infection compared to those with no pre-existing conditions (AOR, 1.75; 95% CI, 1.19–2.56). However, children with chronic lung disease or immunocompromised conditions did not have significantly increased odds of HMPV infection (Table 1).

Age, chronic lung disease, and neurologic/neuromuscular disease were each associated with elevated disease severity among subtyped HMPV cases after adjusting for other characteristics (Table 2). Infants had almost three times higher odds of elevated disease severity compared to older children ≥3 years (AOR, 2.98; 95% CI, 1.42–6.24). Children with chronic lung disease or neurologic/neuromuscular disease had nearly five times higher odds of elevated disease severity (chronic lung AOR, 4.88; 95% CI, 2.39–9.97; neurologic/neuromuscular AOR, 4.80; 95% CI, 2.01–11.47) (Table 2). After controlling for age, sex, race and ethnicity, insurance, pre-existing conditions, and co-infections, there was insufficient evidence to suggest that subgroup was associated with elevated disease severity (Table 2).

**TABLE 2:** Characteristics of subtyped HMPV cases by disease severity level^*a*^

	Overall, *n* = 249^*b*^	HMPV disease severity				Adjusted ordinal logistic regression OR (95% CI)
		Routine discharge from ED/clinic, *n* = 100	Admitted without oxygen support, *n* = 82	Admitted with standard supplemental oxygen, *n* = 47	Death or ICU admission with any outcome, *n* = 20	
Age in years, median (IQR)	1.33 (0.58, 3.00)	2.00 (0.92, 3.00)	1.00 (0.42, 1.92)	2.17 (0.67, 3.75)	1.54 (0.54, 2.29)	NI
Age group, *n* (col%, row%)^*c*^						
Less than 1 year	91 (36.5)	27 (27.0, 29.7)	40 (48.8, 44.0)	16 (34.0, 17.6)	8 (40.0, 8.8)	2.98 (1.42, 6.24)
1 to <2 years	54 (21.7)	22 (22.0, 40.7)	23 (28.0, 42.6)	5 (10.6, 9.3)	4 (20.0, 7.4)	1.69 (0.77, 3.71)
2 to <3 years	38 (15.3)	19 (19.0, 50.0)	8 (9.8, 21.1)	7 (14.9, 18.4)	4 (20.0, 10.5)	1.35 (0.59, 3.12)
3 years or older^*d*^	66 (26.5)	32 (32.0, 48.5)	11 (13.4, 16.7)	19 (40.4, 28.8)	4 (20.0, 6.1)	REF
Sex, *n* (col%, row%)						
Female	108 (43.4)	45 (45.0, 41.7)	37 (45.1, 34.3)	15 (31.9, 13.9)	11 (55.0, 10.2)	0.91 (0.54, 1.52)
Male	141 (56.6)	55 (55.0, 39.0)	45 (54.9, 31.9)	32 (68.1, 22.7)	9 (45.0, 6.4)	REF
Race and ethnicity, *n* (col%, row%)						
Black non-Hispanic	78 (31.7)	52 (53.1, 66.7)	16 (19.8, 20.5)	6 (12.8, 7.7)	4 (20.0, 5.1)	0.20 (0.10, 0.38)
White non-Hispanic	130 (52.8)	36 (36.7, 27.7)	46 (56.8, 35.4)	35 (74.5, 26.9)	13 (65.0, 10.0)	REF
Other non-Hispanic^*e*^	27 (11.0)	8 (8.2, 29.6)	12 (14.8, 44.4)	6 (12.8, 22.2)	1 (5.0, 3.7)	0.66 (0.29, 1.50)
Hispanic	11 (4.5)	2 (2.0, 18.2)	7 (8.6, 63.6)	0 (0, 0)	2 (10.0, 18.2)	1.13 (0.35, 3.66)
Insurance type, *n* (col%, row%)						
Private	79 (33.1)	26 (27.4, 32.9)	30 (37.0, 38.0)	17 (38.6, 21.5)	6 (31.6, 7.6)	REF
Public	149 (62.3)	66 (69.5, 44.3)	49 (60.5, 32.9)	22 (50.0, 14.8)	12 (63.2, 8.1)	0.94 (0.52, 1.67)
Both	5 (2.1)	1 (1.1, 20.0)	0 (0, 0)	4 (9.1, 80.0)	0 (0, 0)	1.30 (0.25, 6.80)
None	6 (2.5)	2 (2.1, 33.3)	2 (2.5, 33.3)	1 (2.3, 16.7)	1 (5.3, 16.7)	1.43 (0.27, 7.59)
HMPV subgroup, *n* (col%, row%)						
A2	103 (41.4)	45 (45.0, 43.7)	31 (37.8, 30.1)	19 (40.4, 18.4)	8 (40.0, 7.8)	REF
B1	56 (22.5)	21 (21.0, 37.5)	19 (23.2, 33.9)	12 (25.5, 21.4)	4 (20.0, 7.1)	1.02 (0.52, 2.00)
B2	90 (36.1)	34 (34.0, 37.8)	32 (39.0, 35.6)	16 (34.0, 17.8)	8 (40.0, 8.9)	1.01 (0.57, 1.78)
Any pre-existing condition, *n* (col%, row%)^*f*^	100 (40.3)	26 (26.3, 26.0)	31 (37.8, 31.0)	30 (63.8, 30.0)	13 (65.0, 13.0)	NI
Pre-existing condition, *n* (col%, row%)						
Chronic lung disease^*g*^	53 (21.4)	18 (18.2, 34.0)	10 (12.2, 18.9)	16 (34.0, 30.2)	9 (45.0, 17.0)	4.88 (2.39, 9.97)
Neurologic/neuromuscular disease^*h*^	24 (9.7)	3 (3.0, 12.5)	8 (9.8, 33.3)	8 (17.0, 33.3)	5 (25.0, 20.8)	4.80 (2.01, 11.47)^*i*^
Immunocompromised condition	3 (1.2)	1 (1.0, 33.3)	1 (1.2, 33.3)	1 (2.1, 33.3)	0 (0, 0)	0.39 (0.04, 3.36)
HMPV cases with multiple viral detections, *n* (col%, row%)	59 (23.7)	27 (27.0, 45.8)	16 (19.5, 27.1)	10 (21.3, 16.9)	6 (30.0, 10.2)	0.95 (0.51, 1.78)
Other viruses detected, *n* (col%, row%)						
Adenovirus	16 (6.5)	6 (6.1, 37.5)	4 (4.9, 25.0)	4 (8.5, 25.0)	2 (10.0, 12.5)	NI
Influenza	3 (1.2)	0 (0, 0)	2 (2.4, 66.7)	1 (2.1, 33.3)	0 (0, 0)	NI
Parainfluenza	7 (2.8)	2 (2.0, 28.6)	2 (2.4, 28.6)	2 (4.3, 28.6)	1 (5.0, 14.3)	NI
Respiratory syncytial virus	2 (0.8)	1 (1.0, 50.0)	1 (1.2, 50.0)	0 (0, 0)	0 (0, 0)	NI
Rhinovirus/enterovirus	36 (14.5)	20 (20.0, 55.6)	8 (9.8, 22.2)	5 (10.6, 13.9)	3 (15.0, 8.3)	NI

^*a*^ OR: (adjusted) odds ratio for elevated illness severity; CI: confidence interval; IQR: inter-quartile range, 25th to 75th percentiles; NI: not included due to pre-specification and/or redundant variable(s) already in model; REF: reference group.

^*b*^ Of 249 cases with known HMPV subgroup, 246 were unique patients (three cases were repeated infections/enrollments).

^*c*^ Column percentages are shown for overall data, and column and row percentages are shown for disease severity levels.

^*d*^ The age group 3 years and older is shown due to the small number of subtyped HMPV cases in the 5 years and older group. The AOR (95% CI) comparing <1 year olds to children 3 to <5 years is 2.85 (1.77, 8.64), which is similar to the AOR comparing <1 year olds to children 3 years and older.

^*e*^ Patients who indicated Asian, Asian American, American Indian, Alaska Native, Native Hawaiian, Pacific Islander, or more than one category were classified as other race due to small sample sizes.

^*f*^ Includes cardiovascular disease, chronic kidney disease, Down syndrome, genetic/metabolic disorders, blood disorders, chronic liver disease, diabetes mellitus, chronic endocrine conditions, chronic lung disease, congenital heart defects, neurologic/neuromuscular disease, and immunocompromised status.

^*g*^ Includes reactive airway disease, chronic lung disease of prematurity, cystic fibrosis, bronchopulmonary dysplasia, and unspecified other lung diseases.

^*h*^ Includes cerebral palsy, seizure disorders, and unspecified other neurologic or neuromuscular disorders.

^*i*^ The adjusted effect of chronic lung disease was not significantly different from the adjusted effect of neurologic/neuromuscular disease, based on a model contrast.

### HMPV seasonality

**Fig 1:**
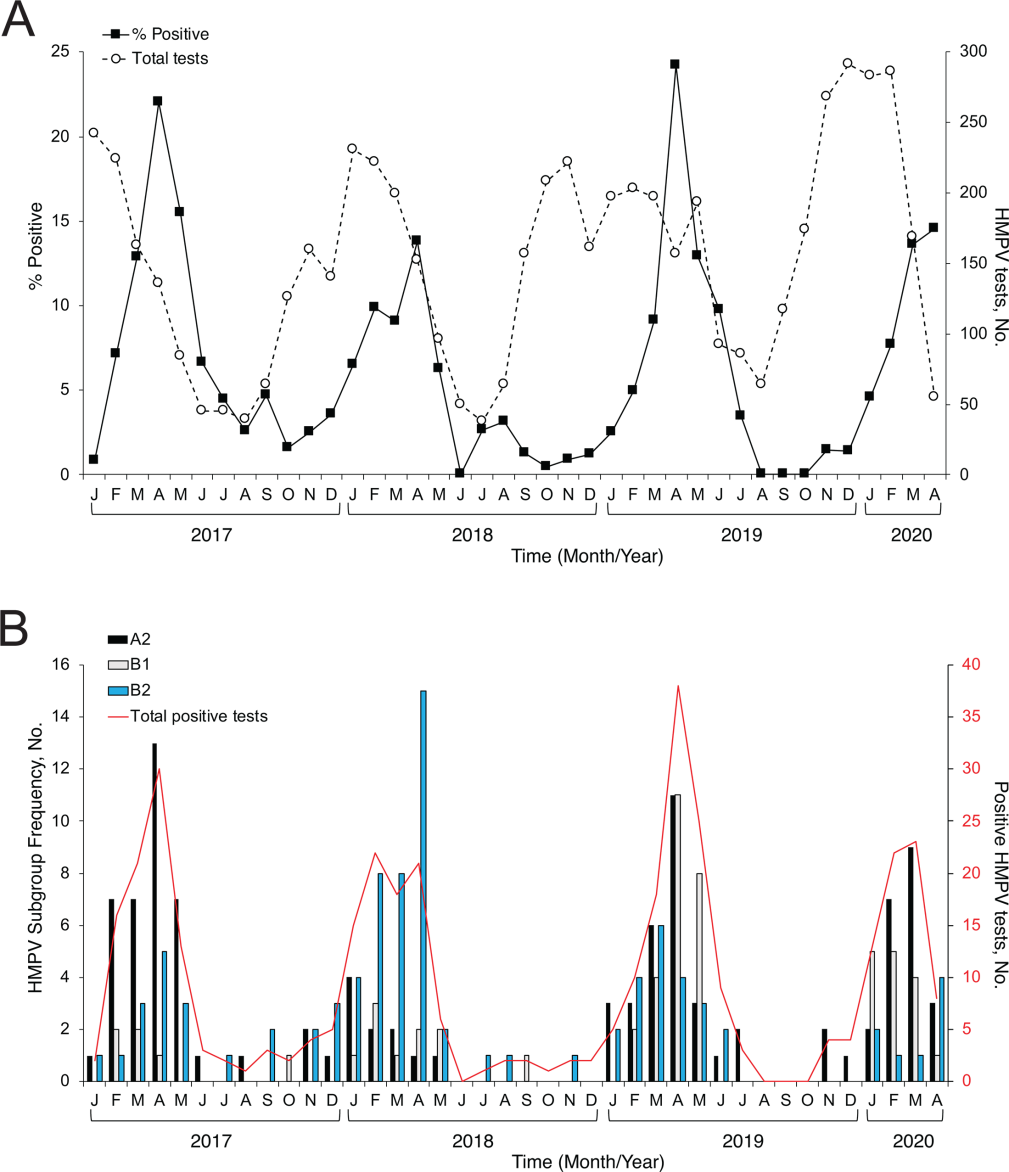
HMPV subgroup frequency and seasonality from 2017 to 2020. (**A**) The percentage of positive HMPV tests (black squares) and total HMPV tests (open circles) are shown by month. The months December 2016 (two positive tests) and August 2020 (one positive test) with indeterminate subgroup are not shown. No HMPV was detected in other months after April 2020. (**B**) HMPV-positive specimens were sequenced to determine subgroup. Subgroup distribution by month is shown for A2 (black bars), B1 (gray bars), and B2 (blue bars). The A1 subgroup was not detected. The total number of HMPV-positive tests is shown by month (red line).

HMPV was more commonly detected in the spring months. HMPV consistently peaked in April every season, with test positivity reaching as high as 24.2% in April 2019 (Fig. 1A), although this pattern was disrupted by the SARS-CoV-2 pandemic in March 2020. There were no positive HMPV samples collected from May to August 2021, except for one detection in August 2020. A2, B1, and B2 viruses co-circulate, and the dominant subgroup varied from season to season (Fig. 1B). The A1 subgroup was not detected, consistent with previous reports that the lineage may have become extinct (14, 47).  

### A2 G gene insertion variants 

**Fig 2:**
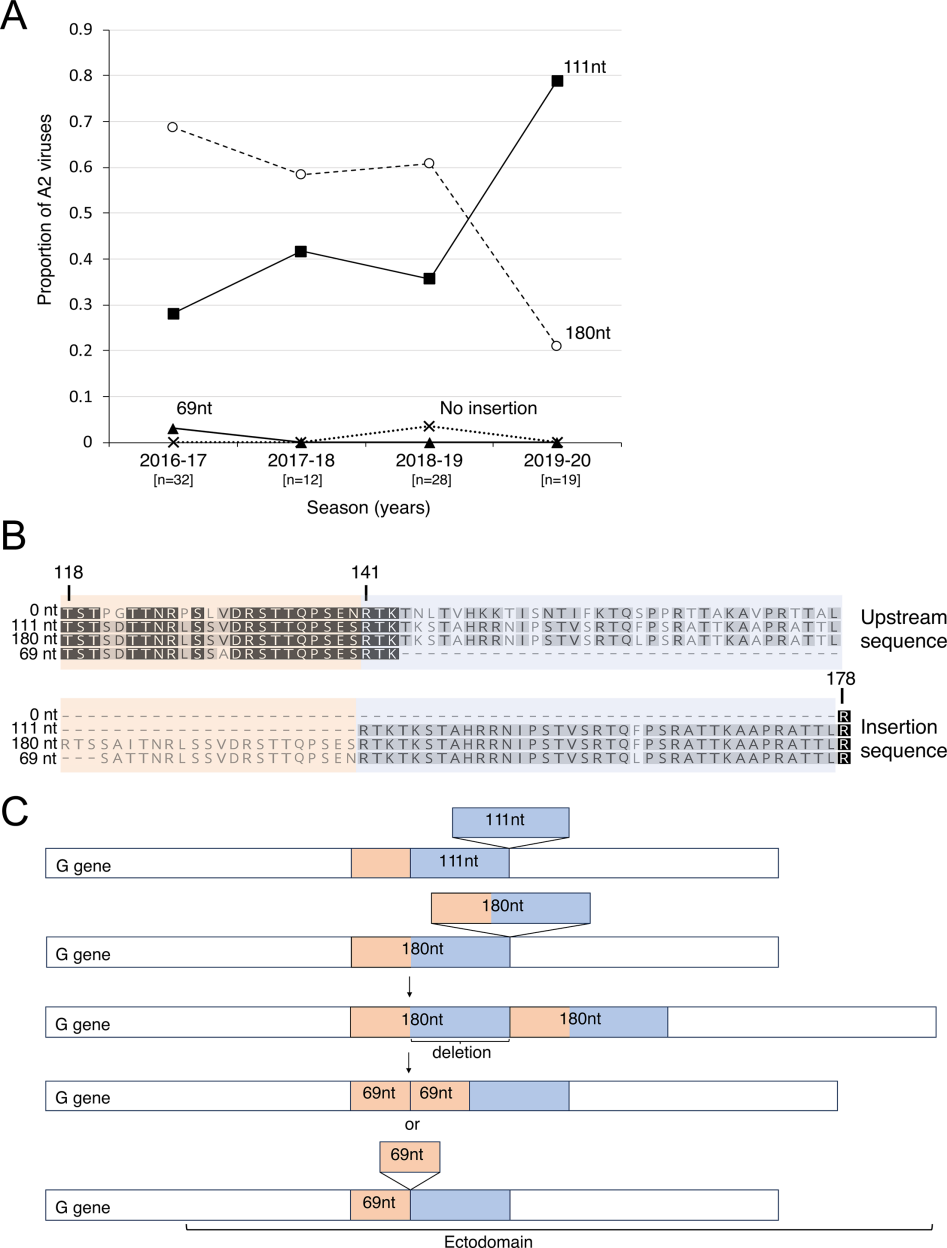
A2 variants with large insertions in the G gene. (**A**) Distribution of A2 G insertion variants over time. The 180-nt variant (open circles) made up the largest proportion of A2 viruses until the 2019–2020 season, when the 111-nt variant (filled squares) became the most predominant detected variant. One virus with no insertion (x markers) and one virus with a 69-nt insertion (filled triangles) were detected during the study period. The total number of A2 genome sequences for each season is shown in square brackets. (**B**) Excerpt of a multiple sequence alignment of G protein consensus sequences shows that the insertions occur in the same location in the ectodomain. The 111-nt variant harbors an insertion that nearly duplicates the upstream 111-nt (37 amino acid) sequence (blue shading). The 180-nt variant has an insertion that nearly duplicates the same 111-nt sequence and an additional 69-nt sequence further upstream (pink shading). The 69-nt variant has a duplication of the same 69-nt sequence in the 180-nt variant. Consensus sequences were constructed by first aligning the whole-genome nucleotide sequences for each variant length (111-nt variant, *n* = 39; 180-nt variant, *n* = 50), followed by extraction of the G gene nucleotide sequences. Geneious Prime was used to generate consensus G nucleotide sequences at a strict 50% threshold. The consensus sequences were translated and aligned with the 0-nt and 69-nt variant G protein sequences. Darker shaded residues indicate higher similarity. Amino acid numbering of the classic strain with no insertion is indicated using vertical lines. (**C**) Cartoon drawn proportionally that illustrates that the variants harbor near-duplications of overlapping regions in the ectodomain. The 69-nt variant could have resulted from a duplication of the 69-nt sequence or a deletion of the 111-nt region from the 180-nt variant.

Analysis of the G gene sequences revealed that nearly all A2 viruses had a 111- or 180-nucleotide (nt) insertion in the ectodomain of the G gene that nearly duplicates the upstream flanking 37 or 60 amino acid residues (Fig. 2). There was only one detection of the classic strain with no insertion. During earlier seasons from 2017 to 2019, the 180-nt variant outnumbered the 111-nt variant; however, during the 2019–2020 season, the proportion of the 111-nt variant rose to nearly 80% of A2 viruses detected. There was also one detection of a novel 69-nt insertion variant that was collected in 2017, but it was not detected again during the study period (Fig. 2A). All three lengths of insertions appeared in the same location. Compared to the 111-nt variant, the 180-nt variant harbors a longer near-duplication that includes the same 111-nt region plus an additional 69 nucleotides further upstream. The 69-nt variant contains an insertion that nearly duplicates only the 69-nt region unique to the 180-nt variant (Fig. 2B and C).

After controlling for age, sex, and pre-existing conditions, there was insufficient evidence to suggest that A2 insertion size was associated with elevated disease severity (Table S4).

### B2 G gene insertion variants

**Fig 3:**
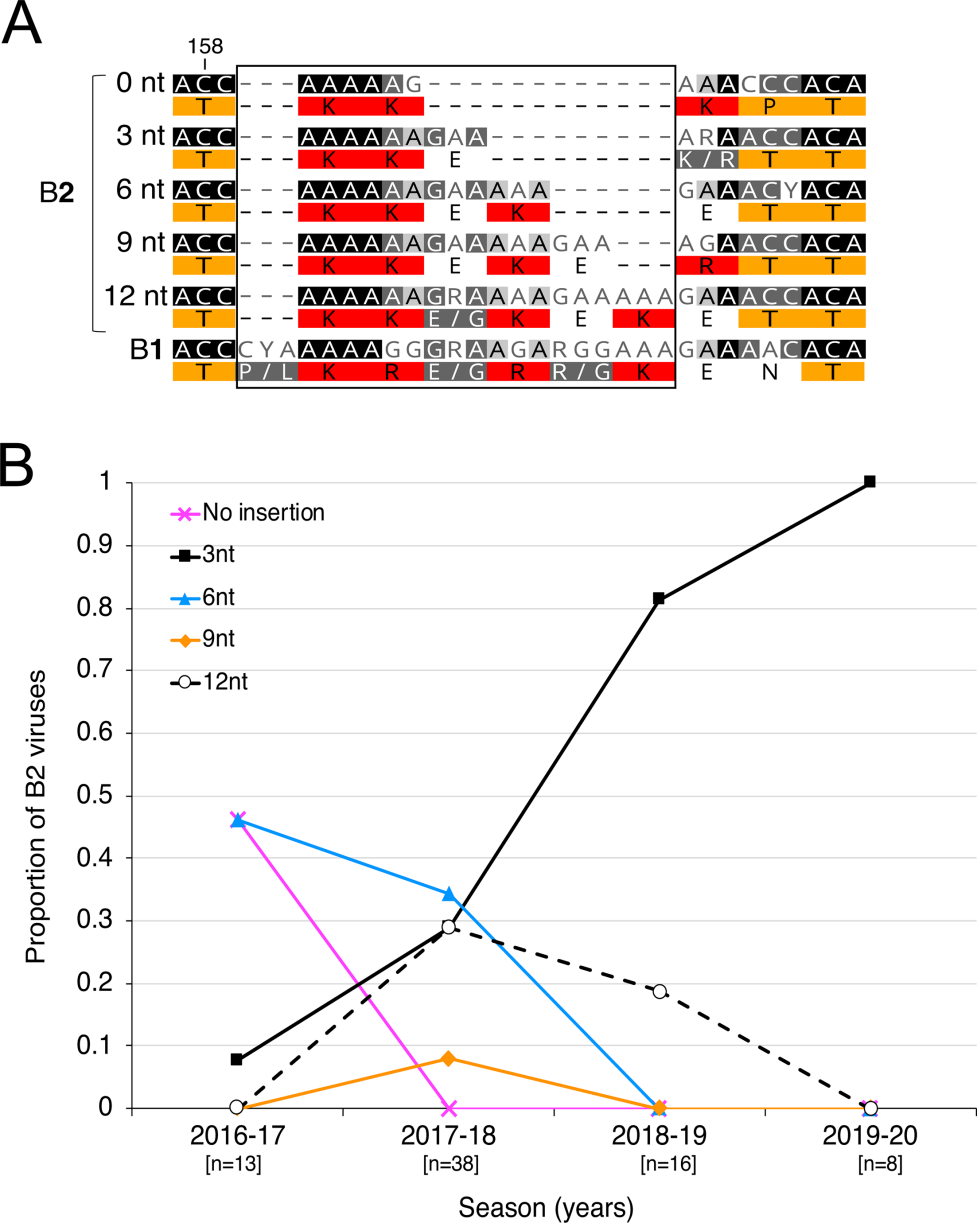
B2 variants with small insertions in the G gene. (**A**) Excerpt of a multiple sequence alignment of G gene consensus sequences showing the B2 insertions (boxed region) aligned to a B1 G sequence that is not present in the classic B2 strain with no insertion. Consensus sequences were constructed by first aligning the whole-genome nucleotide sequences for each variant length (0-nt variant, *n* = 6; 3-nt variant, *n* = 33; 6-nt variant, *n* = 19; 9-nt variant, *n* = 3; 12-nt variant, *n* = 12), followed by extraction of the G gene nucleotide sequences. Geneious Prime was used to generate consensus G nucleotide sequences at a very strict 95% threshold. A multiple nucleotide sequence alignment was made with the consensus sequences, and the encoded amino acid residues are shown under the nucleotides. Darker shaded nucleotides indicate higher similarity. Residues are shaded using the Clustal color scheme. Amino acid numbering of the classic strain with no insertion is indicated using vertical lines. (**B**) Distribution of B2 G insertion variants over time. The classic strain with no insertion (magenta line) was no longer detected after the 2016–2017 season. While several lengths of insertion variants were detected during the 2017–2018 season, the 3-nt variant (black squares) has become the most detected variant since 2018–2019. The total number of A2 genome sequences for each season is shown in square brackets.

**Fig 4:**
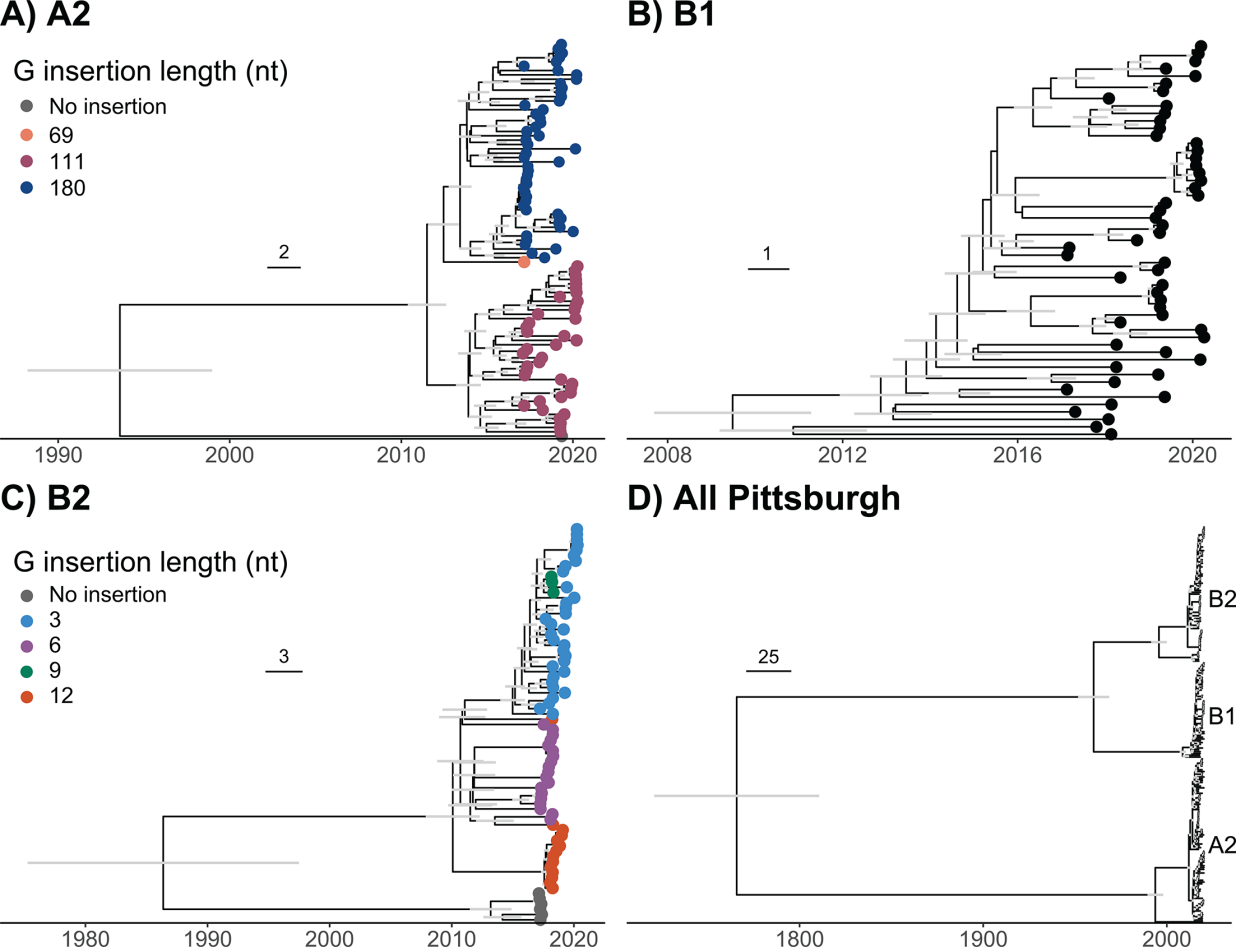
G gene insertion lengths in the A2 and B2 subgroups form distinct clades. Bayesian time-resolved phylogenetic reconstructions were performed using HMPV nucleotide sequence alignments of Pittsburgh samples by subgroup: (**A**) A2; (**B**) B1; (**C**) B2; and (**D**) all Pittsburgh samples together. Tips were colored by size of the nucleotide insertion in the G gene. Gray bars indicate the 95% highest posterior density of the node height for nodes of the phylogeny that had >90% posterior probability. Time in years is indicated on the x-axis. Scale bar indicates number of nucleotide substitutions per site.

We found that many B2 HMPV specimens collected since the 2016–2017 season have novel in-frame insertions of 3, 6, 9, or 12 nucleotides, encoding one to three additional amino acid residues, compared to a B2 reference sequence. The additional residues were predominantly lysine and glutamic acid. These B2 insertion sequences align to a stretch of 15 additional nucleotides in the B1 G gene that are not present in the classic B2 G gene (Fig. 3A). Unlike B2, no B1 viruses harbored any G gene insertions (Fig. 4B). During the 2016–2017 season, almost half of all B2 viruses were the classic strain with no insertion; however, the classic strain was not detected in subsequent seasons during the study period. B2 insertion variants of any size were the only B2 viruses detected by the 2017–2018 season, with the 3-nt variant being the most commonly detected B2 variant by the 2018–2019 season (Fig. 3B).

### Phylogenetic analysis

**Fig 5:**
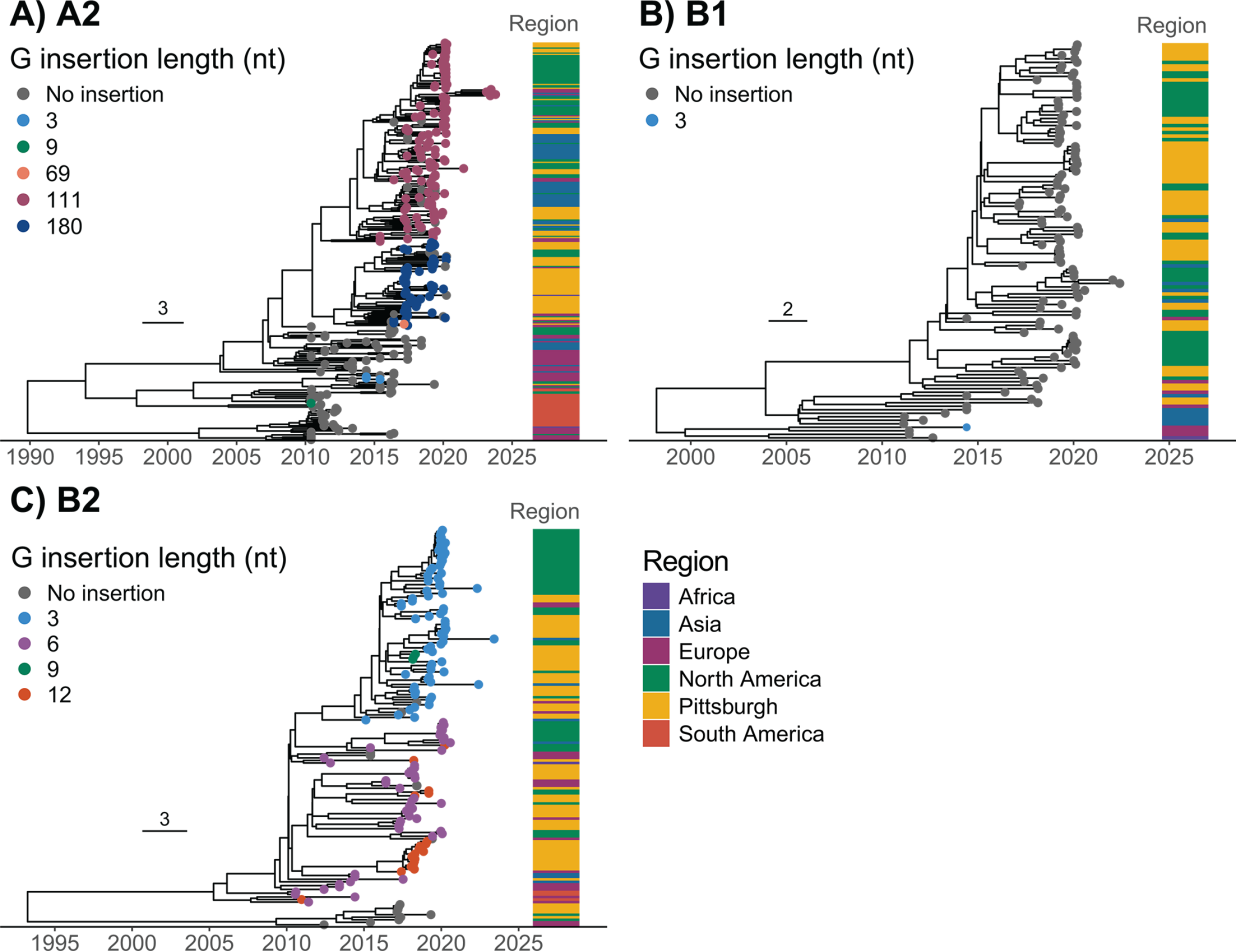
HMPV is globally heterogeneous for place of isolation across subgroups. Bayesian time-resolved phylogenetic reconstructions were performed using HMPV nucleotide sequence alignments of global samples by subgroup: (**A**) A2; (**B**) B1; and (**C**) B2. Tips are colored by insertion length and the geographical region of sample collection. Gray bars indicate the 95% highest posterior density of the node height for nodes of the phylogeny that had >90% posterior probability. Time in years is indicated on the x-axis. The scale bar represents the number of nucleotide substitutions per site.

We performed a phylogenetic analysis of HMPV at the local scale (including only samples collected in Pittsburgh) and at the global scale (including Pittsburgh sequences and publicly available sequences collected globally). Insertion lengths in the A2 and B2 subgroups generally formed distinct phylogenetic clades in both the Pittsburgh (Fig. 4) and global analyses (Fig. 5). The clades for each insertion co-circulated across years for both subgroups. Analysis of viruses sampled globally showed higher resolution of insertion emergence and limited geographic structuring between sequences from different continents (Fig. 5). In the global B2 analysis, the 12-nucleotide insertion emerged independently five times, each time evolving from viruses with the 6-nucleotide insertion (Fig. 5C). Pittsburgh samples were interspersed across each subgroup tree (Fig. 5; Fig. S4). Pittsburgh B1 and B2 viruses formed clades that were related to North American samples, whereas A2 viruses formed more distinct clades of only Pittsburgh-related samples. Samples collected in Pittsburgh contributed nearly half of all available B1 and B2 sequences (46.5% and 47.8%, respectively) and nearly all available A2 180-nt variant whole-genome sequences. To more accurately represent the global circulation of the virus, greater sampling is needed, especially for B1 and B2 viruses, which were sampled at a lower frequency (19.2% and 26.5% of available sequences, respectively).

## DISCUSSION

This genomic epidemiological study contributed 219 full-length HMPV genome sequences collected from 2017 to 2020 and detected HMPV variants with insertions in the ectodomain of the G gene that became the predominant circulating strains in Pittsburgh, PA, by the 2017–2018 season. This suggests that insertions in the G gene may confer a viral fitness advantage. Our exploratory epidemiological analysis, using medical records of all subtyped HMPV samples and the entire patient cohort of nearly 8,000 ARI cases, identified factors associated with HMPV infection and elevated disease severity.

Variants in the A2 subgroup harbored large (69, 111, or 180-nt) near-duplications in the ectodomain of the 660-nt G gene, whereas B2 variants had small (3, 6, 9, or 12-nt) insertions near the same region of the G gene as the A2 G insertions. No insertions were detected in B1 viral genomes. Only one A2 virus with no insertion was detected during the study period, indicating that the insertion variants had become the dominant A2 viruses before the 2016–2017 season. The B2 viruses with no insertion were no longer detected after the 2016–2017 season.

The A2 111- and 180-nt variants have been previously detected elsewhere (14–19). We observed that the 180-nt variant was more common than the 111-nt variant earlier in the study period, but then the 111-nt later overtook the 180-nt variant during the 2019–2020 season. These results are consistent with smaller studies that reported dynamics between these two variants (14, 19–21).

The A2 G insertion sequences we detected are predicted to contain additional O-glycosylation sites, similar to the insertion sequences found in other studies (15–17). These additional O-glycosylation sites could enhance binding to host cells during infection (48). RSV, which is related to HMPV, also has two sizes of G insertion variants that have become the dominant strains circulating globally, and one variant was shown to have enhanced attachment compared to wild-type virus (49–51). We speculate that the 111-nt variant could have outcompeted the 180-nt variant because the excessive O-glycosylation sites in the 180-nt variant may have enhanced attachment to the point of hindering viral budding and subsequent infection, relative to the 111-nt variant. The 111-nt variant may have a moderate level of enhanced attachment, which could have resulted in a comparative advantage over the 180-nt variant. Alternatively, it is possible that the insertions with additional potential glycosylation sites could enhance “glycan shielding” to evade the host immune response (48). G does not appear to elicit neutralizing antibodies and is thus unlikely to be subject to immune pressure to escape antibody recognition (52–54). Nevertheless, G is an important virulence factor, since infection with a recombinant HMPV virus lacking G was strongly attenuated in non-human primates (55). It has been hypothesized that G sterically shields antigenic epitopes on the fusion protein F; therefore, insertions and additional glycosylation that increase the size of G may enhance its potential to shield F from neutralizing antibodies (56, 57).

The identification of a novel A2 69-nt insertion variant in our study raises an intriguing possibility that the insertion sequences could function as modular units, since the 180-nt insertion is the combination of the 69-nt and 111-nt sequences (Fig. 2C). More sampling is needed to determine whether the 69-nt variant emerged from the 180-nt variant following a deletion of the 111-nt region or as a straightforward duplication of the upstream 69-nt region.

To our knowledge, this is the first detection of B2 variants with insertions in the G gene ectodomain in the USA. B2 G insertions were previously detected in Spain (21, 56), but the genetic characteristics and frequencies of different insertion lengths were not assessed. Interestingly, we found that the B2 G insertions of alternating lysine and glutamate residues align to a B1 subgroup G sequence containing a lysine-arginine-glutamate/glycine motif unique to the B1 G gene (58). Recently collected B2 variants have insertions “filling in” to copy this B1 motif with similar residues, suggesting convergent evolution between the B1 and B2 G genes. Although the function of this motif is unclear, the additional positively charged lysines in the B2 insertion could enhance viral attachment to host cells. Glutamate could form salt bridges with lysine to locally stabilize this region, since the G ectodomain appears to be unstructured (57). Though the 3-nt variant became the predominant circulating B2 virus in our sample collection, more sampling is needed to parse the dynamics between the different lengths of B2 insertion variants.

Furthermore, our whole-genome phylodynamics analysis showed that the different A2 and B2 insertion variants form distinct clades and that there is limited geographical structuring, although sampling is heterogeneous across regions. Therefore, more genomic surveillance is needed to determine whether G insertions confer a viral fitness advantage that leads to continued propagation and contributes to global spread.

Multiple previous studies have evaluated whether any subgroup is associated with elevated disease severity. These studies have conflicting results, with some concluding that severity is associated with A1/A2 (56, 59), B1/B2 (60), or no particular subgroup (14, 61, 62). Using medical records associated with all subtyped HMPV samples, we did not detect a statistically significant difference in elevated disease severity between the subgroups. Compared to other studies, our study had a larger HMPV-positive sample size and the ability to control for variables, such as age and pre-existing conditions, which we interpret to contribute the primary effects on elevated disease severity. Having either a pre-existing chronic lung or neurologic/neuromuscular disease was associated with five times greater odds of elevated disease severity. Although a larger proportion of patients at every level of elevated disease severity had the A2 180-nt variant compared to the 111-nt, this difference was not statistically significant at this sample size.

Previous studies offer conflicting conclusions on the relationship between co-infections and disease severity, with some studies finding no association (2, 63–65) and others concluding an association between HMPV-RSV co-infection and certain severity metrics (66–68). Our analysis did not detect an independent association between elevated disease severity and co-infection status among subtyped HMPV samples (Table 2). However, the sample size of analyzed cases with viral co-detections was small. Larger studies of HMPV cases are needed to examine the association between co-infections and elevated disease severity, stratified by co-detected virus.

Our exploratory epidemiological analysis also revealed that age, insurance type, and neurologic/neuromuscular disease were associated with HMPV infection. HMPV infections are rarely detected in asymptomatic children (2, 69), and our study population was drawn from children who sought medical care for ARI symptoms. Our finding that all younger age groups have at least twice greater odds of medically attended HMPV infection, compared to school-age children, likely stems from a higher probability of primary infection in younger children with more naïve immune systems or smaller airways, which may prompt parents to seek medical care. Relatedly, this study found that infants, compared to children 3 years or older, had nearly three times greater odds of elevated disease severity, possibly due to infants having smaller airways that are more easily obstructed by inflammation caused by a primary HMPV infection.

Moreover, children with no insurance or both private and public insurance had at least twice greater odds of HMPV infection compared to those with private insurance. This could be due to a complex combination of access to preventative healthcare and/or socioeconomic factors. Though investigating underlying factors was outside the scope of this study, one possible explanation is that insurance type could be related to parents having employment that does not provide adequate private health insurance. These jobs could intrinsically involve more human contact and therefore increase disease exposure to the children’s households. An association between insurance and other respiratory viral infections, such as SARS-CoV-2, has also been previously published, although the effect size is much greater in this study (70). In Pennsylvania, children with severe health conditions can qualify for public insurance, allowing them to be covered by both public and private insurance plans. However, even after controlling for other covariates including comorbidities, children with both public and private insurances were still at significantly higher odds of HMPV infection compared to those with only private insurance.

Our data indicated that children with neurologic/neuromuscular conditions had 75% greater odds of HMPV infection after adjusting for all other covariates. Chronic lung disease was not significantly associated with increased odds of HMPV infection. There is almost no overlap between those with chronic lung and neurologic/neuromuscular disease in the analyzed patient cohort (only five have both conditions). There are few studies on HMPV and neurologic/neuromuscular conditions, and most of them are case reports or surveys evaluating whether HMPV infection causes encephalopathy or seizure (71–73). Other studies assessed HMPV disease severity and concluded that pre-existing neurologic/neuromuscular conditions were associated with more severe disease, which is consistent with our findings (1, 74, 75). However, pre-existing neurologic/neuromuscular conditions have not been well established as a factor associated with HMPV infection. A possible explanation for this association is that impaired airway clearance or pulmonary reserve could increase vulnerability to HMPV infection. This finding warrants further follow-up with a larger sample size.

### Strengths and limitations

The strengths of this study include its prospective study design, with human specimens systematically collected from outpatients and inpatients, along with corresponding patient data from 2016 to 2021, allowing investigation of disease severity and adjustment for variables such as co-infections and comorbidities. To our knowledge, this is the largest prospective population-based genomic epidemiological study of HMPV, analyzing nearly 8,000 ARI cases and contributing 219 new whole-genome HMPV sequences to the existing 604 complete genomes available on NCBI Virus (76).

The study also had some limitations. Patient enrollment only captured pediatric ARI cases that presented to outpatient clinics, the emergency department, or were inpatients in the Pittsburgh metropolitan area, which limits generalizability. The HMPV variant frequencies observed in our Pittsburgh sample collection do not necessarily reflect circulation patterns in other US geographical regions. Not all HMPV-positive samples could be sequenced or subtyped due to inadequate levels of intact viral RNA, which could limit the representation of some subgroups or insertion lengths. Because the G insertions are near-duplications and can be misaligned or omitted during genome assembly (56), we verified insertions detected in our Pittsburgh samples using Sanger sequencing. However, it was not feasible to verify insertions we detected in the global sequences used in our phylogenetics analysis.

### Conclusions

This study identified patient factors associated with infection and disease severity and determined that HMPV G insertion variants were the predominant A2 and B2 viruses circulating in Pittsburgh by the 2017–2018 season. Genomic surveillance and further investigation to define the role of G insertions in viral fitness are needed to better understand HMPV evolution and associated clinical outcomes.

## Data Availability

All genome sequences generated in this study were submitted to GenBank as Bioproject PRJNA1258959 with accession numbers PV796784 to PV797002. Raw sequence reads were uploaded to the Sequence Read Archive (SRA) with accession numbers SRR33431103 to SRR33431321. A complete list of accession numbers for sequences used in the phylodynamics analysis is provided in Table S5. Deidentified human subjects data may be made available in accordance with data use agreements and regulations upon request to the investigative team.

## SUPPLEMENTAL MATERIAL

Supplemental Figures mbio.02619-25-s0001.pdf Figures S1 to S4.

Supplemental Tables mbio.02619-25-s0002.pdf Tables S1 to S4

Table S5 mbio.02619-25-s0003.xlsx Accession numbers and associated information for all HMPV sequences used in the phylodynamics analysis.

ASM does not own the copyrights to Supplemental Material that may be linked to, or accessed through, an article. The authors have granted ASM a non-exclusive, world-wide license to publish the Supplemental Material files. Please contact the corresponding author directly for reuse.
