# Supplementary material for: Emergence and epidemiology of dominant variants of human metapneumovirus in the United States between 2016 and 2021
Source: mBio. 2026 Jan 12;17(2):e02619-25. doi: 10.1128/mbio.02619-25 (PMC12892982; doi:10.1128/mbio.02619-25)
Supplement: Supplemental Figures — Figures S1 to S4. [file mbio.02619-25-s0001.pdf]

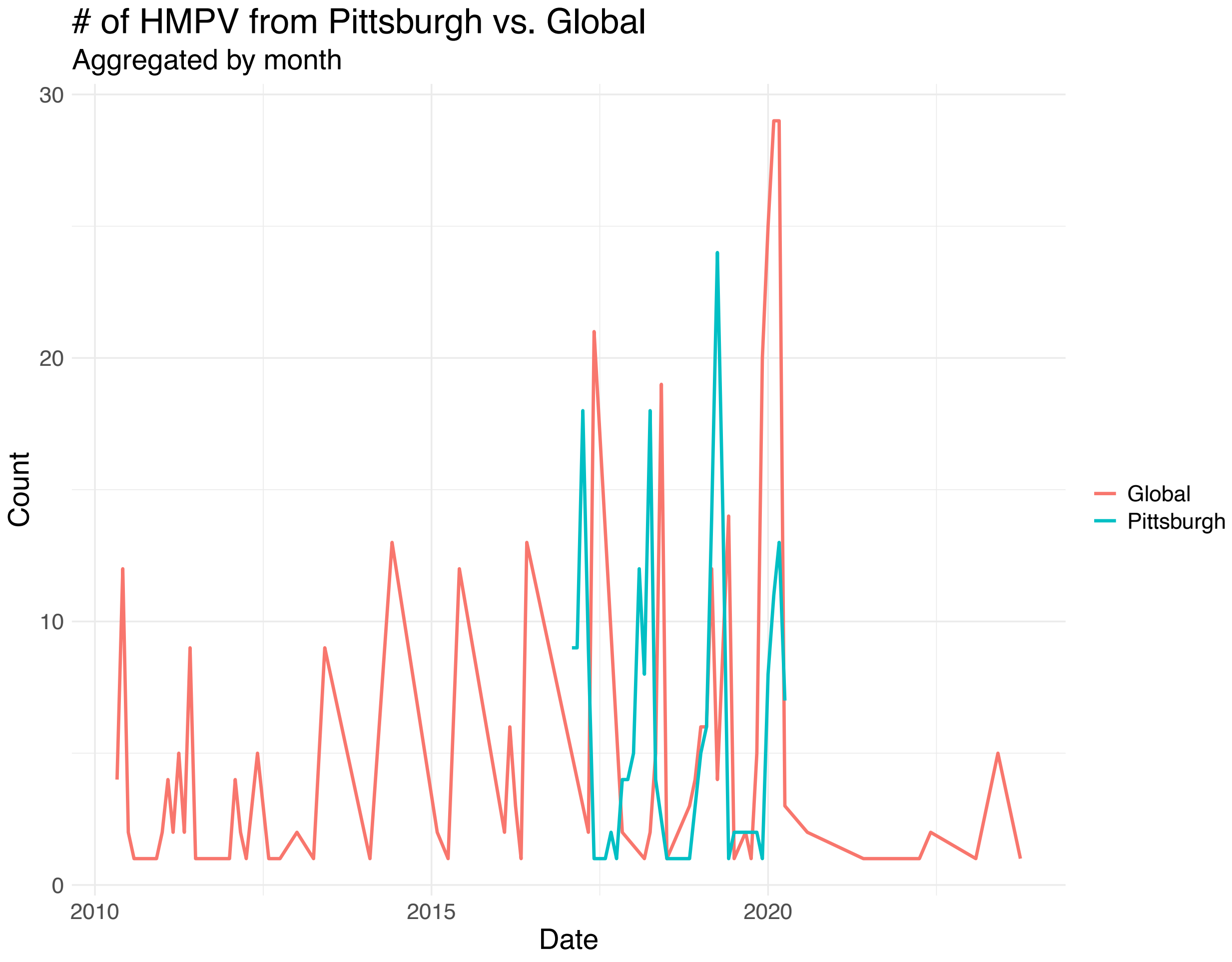

Figure S1. Number of HMPV full-length genome sequences from Pittsburgh generated by this study versus globally sampled sequences from NCBI Virus from January 1, 2010 to August 13, 2024.

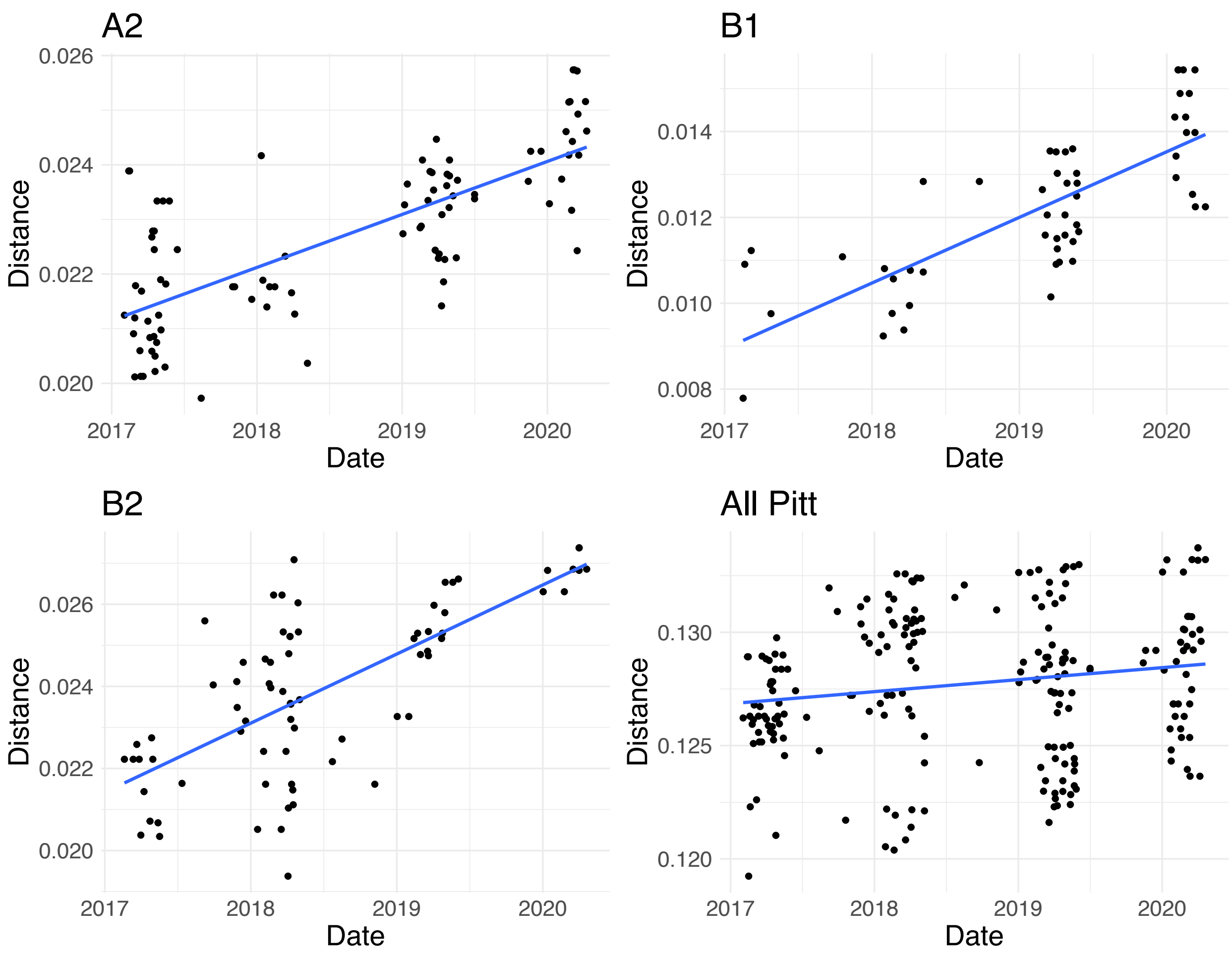

Figure S2. Root-to-tip regression results from TempEst v1.5.3 for Pittsburgh HMPV sequences generated in this study.

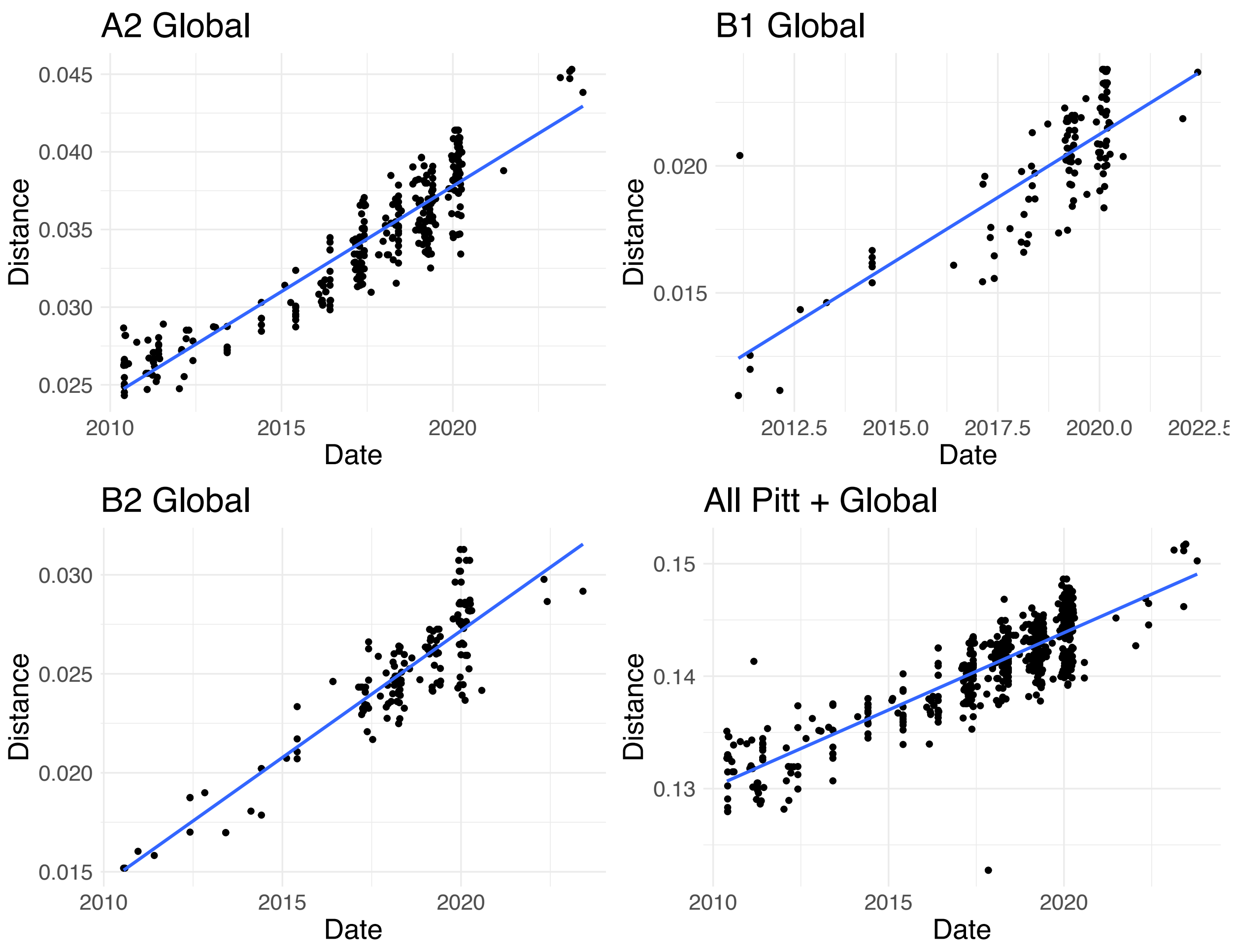

Figure S3. Root-to-tip regression results from TempEst v1.5.3 for global HMPV sequences from NCBI Virus.

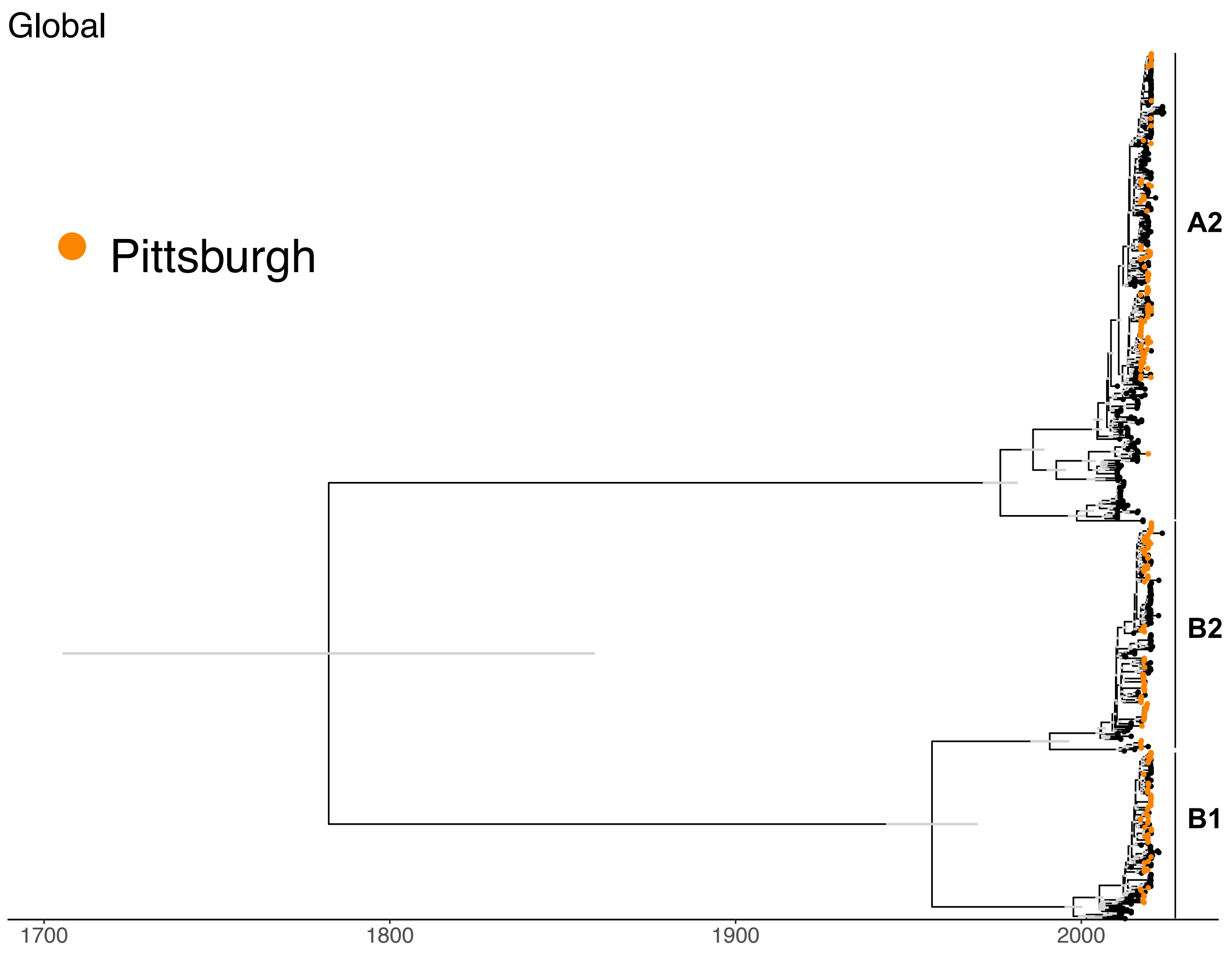

Figure S4. Bayesian time-resolved phylogenetic reconstruction using a nucleotide sequence alignment of all available whole genome sequenced HMPV samples between January 1, 2010 and August 13, 2024. Tips corresponding to samples from Pittsburgh are colored and subgroups are labeled. Grey bars indicate the 95% HPD of the node height for nodes of the phylogeny that had > 90% posterior probability. Time in years is indicated on the x-axis.
