# Supplementary material for: Emergence and epidemiology of dominant variants of human metapneumovirus in the United States between 2016 and 2021
Source: mBio. 2026 Jan 12;17(2):e02619-25. doi: 10.1128/mbio.02619-25 (PMC12892982; doi:10.1128/mbio.02619-25)
Supplement: Supplemental Tables — Tables S1 to S4 [file mbio.02619-25-s0002.pdf]

Table S1. Primer sequences and expected amplicon sizes.

Amplicon	Primer Name	Sequence (5' → 3')	Amplicon Size (bp)
1	hMPV1_F	GGGACAAATAAAAATGTCTCTTCA	4125
	hMPV1_F2	GGGGAARCATGCTATATTAAAAG	
	hMPV1_R	CTTCCTGTGCTRACYTTRCA	
2	hMPV2_F	ACAGCAGCRGGRATYAATGT	4010
	hMPV2_F3	GWTCMWACATGCCRACATCTG	
	hMPV2_R	TAGTACTGAAYTGAGCATGYTCAG	
3	hMPV3_F	AACTGTTAACATGGAAAGATGTGATG	3229
	hMPV3_R	TAAGCTGGAACWGAWGCTG	
4	hMPV4_F	TCAATAGGGAGTCTRTGTCARGAA	3675
	hMPV4_F3	GGTCATAAACTCAAAGAAGGTG	
	hMPV4_R	GRCAAAAAAACCGTATACATYC	

Table S2. Pittsburgh genome sequences modified due to suspected sequencing errors or assembly anomaly that led to frameshifts.

Sample name	Subgroup	Modification
EP1R-02180	A2	Removed 1nt insertion in the N gene that resulted in frameshift and protein truncation
EP2R-02946	B1	Removed 1nt insertion in the M gene that resulted in frameshift and protein truncation
EP1R-00769	B2	Removed 71 nt insertion in the N gene that resulted in frameshift and protein truncation
EP1R-02295	B2	Removed 1 nt insertions in the M and F genes that resulted in frameshifts and protein truncation
EP2R-00490	B2	Removed 1 ambiguous base insertion in the N gene

Table S3. G insertion variant annotations for global sequences with frameshifts or other anomalies in the G gene due to suspected sequencing errors. Sequences were not modified in the phylogenetic analysis.

Accession	Subgroup	G insertion size	Reason for annotation	Annotation on phylogeny:
OL794386	A2	181	Frameshift leads to early stop codon in the insertion	180-nt
PP947671	A2	104	Has 104-nt insertion but also has upstream 16nt "deletion" with early stop codon	0-nt
PP947655	A2	66	Insertion mostly contains ambiguous bases (Ns)	0-nt
OL794430	A2	1	Frameshift leads to early stop codon	0-nt
KY474529	A2	1	1-nt insertion in a string of Ns. Also a missing base (C) upstream, so no net insertion	0-nt
OL794402	B2	4	Frameshift leads to early stop codon	0-nt
OL794432	B2	1	Frameshift leads to early stop codon	0-nt
OL794475	B2	1	Frameshift leads to early stop codon	0-nt
OL794441	B2	5	Frameshift leads to early stop codon	0-nt

Table S4. Characteristics of cases with A2 insertion variants by disease severity level

	Overall, n=89[a]	HMPV Disease Severity				Adjusted Ordinal Logistic Regression OR (95% CI)
		Routine Discharge from ED/Clinic, n=38	Admitted without Oxygen Support, n=26	Admitted with Standard Supplemental Oxygen, n=18	Death or ICU Admission with any outcome, n=7	
**Age in years**, median (IQR)	1.33 (0.67, 2.33)	1.13 (0.75, 2.00)	1.00 (0.42, 1.92)	3.17 (1.00, 4.67)	1.67 (0.58, 2.00)	NI
**Age group**, n (col%, row%)[b]						
Less than 1 year	31 (34.8)	14 (36.8, 45.2)	10 (38.5, 32.3)	4 (22.2, 12.9)	3 (42.9, 9.7)	0.52 (0.15, 1.76)
1-2 years	27 (30.3)	13 (34.2, 48.1)	10 (38.5, 37.0)	3 (16.7, 11.1)	1 (14.3, 3.7)	0.33 (0.10, 1.07)
2-3 years	13 (14.6)	7 (18.4, 53.8)	3 (11.5, 23.1)	1 (5.6, 7.7)	2 (28.6, 15.4)	0.33 (0.08, 1.47)
3 years or older	18 (20.2)	4 (10.5, 22.2)	3 (11.5, 16.7)	10 (55.6, 55.6)	1 (14.3, 5.6)	REF
**Sex**, n (col%, row%)						
Female	40 (44.9)	20 (52.6, 50.0)	10 (38.5, 25.0)	5 (27.8, 12.5)	5 (71.4, 12.5)	1.05 (0.45, 2.47)
Male	49 (55.1)	18 (47.4, 36.7)	16 (61.5 32.7)	13 (72.2, 26.5)	2 (28.6, 4.1)	REF
**Any pre-existing condition**, n (col%, row%)[c]	33 (37.5)	9 (24.3, 27.3)	9 (34.6, 27.3)	12 (66.7, 36.4)	3 (42.9, 9.1)	2.25 (0.86, 5.84)
**HMPV A2 insertion size**, n (col%, row%)						
111nt	39 (43.8)	19 (50.0, 48.7)	10 (38.5, 25.6)	8 (44.4, 20.5)	2 (28.6, 5.1)	REF
180nt	50 (56.2)	19 (50.0, 38.0)	16 (61.5, 32.0)	10 (55.6, 20.0)	5 (71.4, 10.0)	1.37 (0.62, 3.05)

Abbreviations

OR: (Adjusted) Odds ratio for elevated illness severity
CI: Confidence interval
IQR: Inter-quartile range, 25th to 75th percentiles
NI: Not included due to pre-specification and/or redundant variable(s) already in model
REF: Reference group

Footnotes

[a]All were unique patients
[b]Column percentages are shown for overall data, and column and row percentages are shown for disease severity levels.
[c]Includes cardiovascular disease, chronic kidney disease, Down Syndrome, genetic/metabolic disorders, blood disorders, chronic liver disease, diabetes mellitus, chronic endocrine conditions, chronic lung disease, congenital heart defects, neurologic/neuromuscular disease, and immunocompromised status
